# Fecal short chain fatty acids modify therapeutic effects of sleeve gastrectomy

**DOI:** 10.3389/fendo.2023.1277035

**Published:** 2023-11-07

**Authors:** Chongrong Shen, Yanru Chen, Qiaoling Wang, Yingkai Sun, Huibin Lin, Mengshan Ni, Yufei Chen, Ling Zhang, Jiabin Jin, Xiayang Ying, Yuyao Zou, Xiaolin Wang, Yaorui Ye, Miaomiao Yuan, Nan Yin, Zhiwen Cao, Yifei Zhang, Weiqiong Gu, Weiqing Wang, Guang Ning, Jiqiu Wang, Shaoqian Zhao, Jie Hong, Ruixin Liu

**Affiliations:** ^1^ Department of Endocrine and Metabolic Diseases, Shanghai Institute of Endocrine and Metabolic Diseases, Ruijin Hospital, Shanghai Jiao Tong University School of Medicine, Shanghai, China; ^2^ Shanghai National Clinical Research Center for Metabolic Diseases, Key Laboratory for Endocrine and Metabolic Diseases of the National Health Commission of the PR China, Shanghai National Center for Translational Medicine, Shanghai, China; ^3^ Pancreatic Disease Center, Department of General Surgery, Ruijin Hospital, Shanghai Jiao Tong University School of Medicine, Shanghai, China; ^4^ CAS Key Laboratory of Separation Science for Analytical Chemistry, Dalian Institute of Chemical Physics, Chinese Academy of Sciences, Dalian, China

**Keywords:** bariatric surgery, short chain fatty acids, obesity, weight loss, sleeve gastrectomy

## Abstract

**Aims:**

We aimed to investigate changes of fecal short chain fatty acids (SCFAs) and their association with metabolic benefits after sleeve gastrectomy (SG). Specifically, whether pre-surgery SCFAs modify surgical therapeutic effects was determined.

**Methods:**

62 participants with measurements of fecal SCFAs and metabolic indices before and 1, 3, 6 months after SG were included. Changes of fecal SCFAs and their association with post-surgery metabolic benefits were calculated. Then, participants were stratified by medians of pre-surgery fecal SCFAs and modification effects of pre-surgery fecal SCFAs on surgical therapeutic effects were investigated, through calculating interaction of group by surgery.

**Results:**

Fecal SCFAs were markedly changed by SG. Changes of propionate and acetate were positively correlated with serum triglycerides and total cholesterol, respectively. Notably, high pre-surgery fecal hexanoate group showed a better effect of SG treatment on lowering body weight (P=0.01), BMI (P=0.041) and serum triglycerides (P=0.031), and low pre-surgery fecal butyrate had a better effect of SG on lowering ALT (P=0.003) and AST (P=0.019).

**Conclusion:**

Fecal SCFAs were changed and correlated with lipid profiles improvement after SG. Pre-surgery fecal hexanoate and butyrate were potential modifiers impacting metabolic benefits of SG.

## 1 Introduction

Obesity has reached pandemic proportions worldwide in recent decades, and bariatric surgery is the most effective treatment for reducing body mass index (BMI) in people with clinically severe obesity (1). Within these individuals, bariatric surgery offers a sustained weight loss, accompanied with improving obesity-related comorbidities including hyperglycemia, hypertension, and hyperlipidemia (2). However, the efficacy of bariatric surgery-induced metabolic benefits may vary considerably between individuals and the underlying factors remain largely unclear (3, 4).

Mounting evidence suggests that gut microbiota is closely related to obesity and other metabolic diseases in rodent models and humans (5, 6). In general, individuals with obesity show decreased bacterial diversity (7) and gene richness (8, 9), while these changes were largely restored after bariatric surgery (10). Predictive role of gut microbiota in therapeutic effects of bariatric surgery are also suggested by previous studies. For example, Prevotella-to-Bacteroides ratio is significantly lower in patients with excess weight loss less than 50% (4). Gut microbiota exerts its function mainly through different metabolites, however, what kind of metabolites derived from the intestinal microbiota can impact the metabolic benefits after bariatric surgery has scarcely been studied. Short chain fatty acids (SCFAs), are well-known end products of the intestinal microbial fermentation of indigestible dietary components (11), and have been demonstrated to play key functional roles in regulating metabolic homeostasis (12, 13). On the one hand, the extra energy from the SCFAs has been estimated to account for at least 10% of the overall energy intake in adult humans on a westernized diet, with acetate as the main energy source (14). Once in the liver, acetate and butyrate are converted to Acetyl-coA which enters the tricarboxylic acid cycle and eventually generate ATP and NADH (15). Propionate is metabolized to propionyl-CoA and is converted to succinyl-CoA and consequently generates glucose (15). As a matter of fact, propionate has been widely used in diets for dairy cows and sheep to increase glucose concentration in milk (16). On the other hand, SCFAs can be beneficial for metabolic health in some cases. In a human study, an acute administration with inulin-propionate ester significantly increases PYY and GLP-1 secretion, and reduces food intake by a mean reduction of 13.8% (17). In animal models of obesity and type 2 diabetes, oral administration of acetate, butyrate and propionate (18) decrease the accumulation of lipids in the liver and improve glucose tolerance. These studies suggest the multiple roles of SCFAs in the metabolic regulation. However, the influence of fecal SCFAs on the impact of bariatric surgery on metabolic benefits has not been investigated.

The two most common bariatric surgeries are Roux-en Y gastric bypass (RYGB) and sleeve gastrectomy (SG) with different gastrointestinal operations. Compared with RYGB, SG procedure is simpler with fewer operative risk and less post-operative complications and is more widely applied (19–21). Two previous studies using samples including both RYGB and SG revealed a decrease in acetate, propionate and butyrate at 4 to 12 months after surgeries (22, 23), and the absolute levels of branched SCFAs are significantly increased only in one study (23). However, both studies revealing changes in fecal SCFAs after bariatric surgery are mainly from RYGB, and small sample size of SG with limited follow-up time points (22, 23). As the two bariatric surgery types operate at different gastrointestinal parts, analysis using a combination of RYGB and SG is hard to see the effects of each surgy type individually. A comprehensive understanding of SCFAs changes at different time points after SG is still lacking. In addition, most of the participants in previous studies are European (23) or American (22), without data in Chinese people (22, 23). What’s more, no studies have discussed the impact of fecal SCFAs on metabolic response to bariatric surgery.

Here we comprehensively compared the profiles of fecal SCFAs in 62 subjects with obesity before and at 1, 3, 6 months after SG intervention and investigated the correlation between changes in fecal SCFAs and metabolic indices (body weight, BMI, lipid profiles, liver function, fasting glucose and insulin, blood pressure). Moreover, we classified the participants into two groups based on the medians according to baseline fecal concentrations of each SCFA, and analyzed the influence of pre-surgery fecal SCFAs on metabolic response to SG treatment through calculating the interaction of group by surgery, revealing that pre-surgery fecal hexanoate and butyrate can impact metabolic improvements of SG intervention, providing potential factors guiding post-surgery metabolic benefits.

## 2 Materials and methods

### 2.1 Cohorts

Participants with obesity (body mass index [BMI] ≥ 30 kg/m2) were recruited in the specialized obesity outpatient clinic of Ruijin Hospital, Shanghai Jiao Tong University School of Medicine, from the Genetics of Obesity in Chinese Young study established by Ruijin Hospital and registered at Clinical Trials. Gov (Clinical trial reg. no. NCT01084967 and NCT02653430, http://www.clinicaltrials.gov/) (10). In this study, we evaluated 62 subjects with obesity who underwent SG meeting the criteria according to “the Chinese Guidelines for Surgical Treatment of Obesity and Type 2 Diabetes Mellitus (2019 edition)” formulated by the Chinese Society for Metabolic & Bariatric Surgery (24). Only omnivorous individuals were included. Exclusion criteria were: (1) history of usage of any antibiotic consumption within 3 months before sample collection; (2) history of intake of any food containing probiotics such as yogurt within 7 days before sample collection. Surgeries were performed by the same group, with standardization of the processes and technique.

In our study, all patients were given the same advice on their diets before or after the operation, according to “the Chinese Guidelines for Surgical Treatment of Obesity and Type 2 Diabetes Mellitus (2019 edition)” formulated by the Chinese Society for Metabolic & Bariatric Surgery (24): (1) Low-calorie diets (recommended daily calorie intake: 1400 kcal/day) were given from 10 days before the operation in the hospital; (2) From 1 to 7 days after the operation, bland and liquid food was given according to the patients' condition; (3) When all the patients were discharged from the hospital, low-sugar, low-fat, decaffeinated semi-liquid and soft foods were recommended within one month after surgery. All the patients complied well with the recommendation as self-reported.

**Figure 1:**
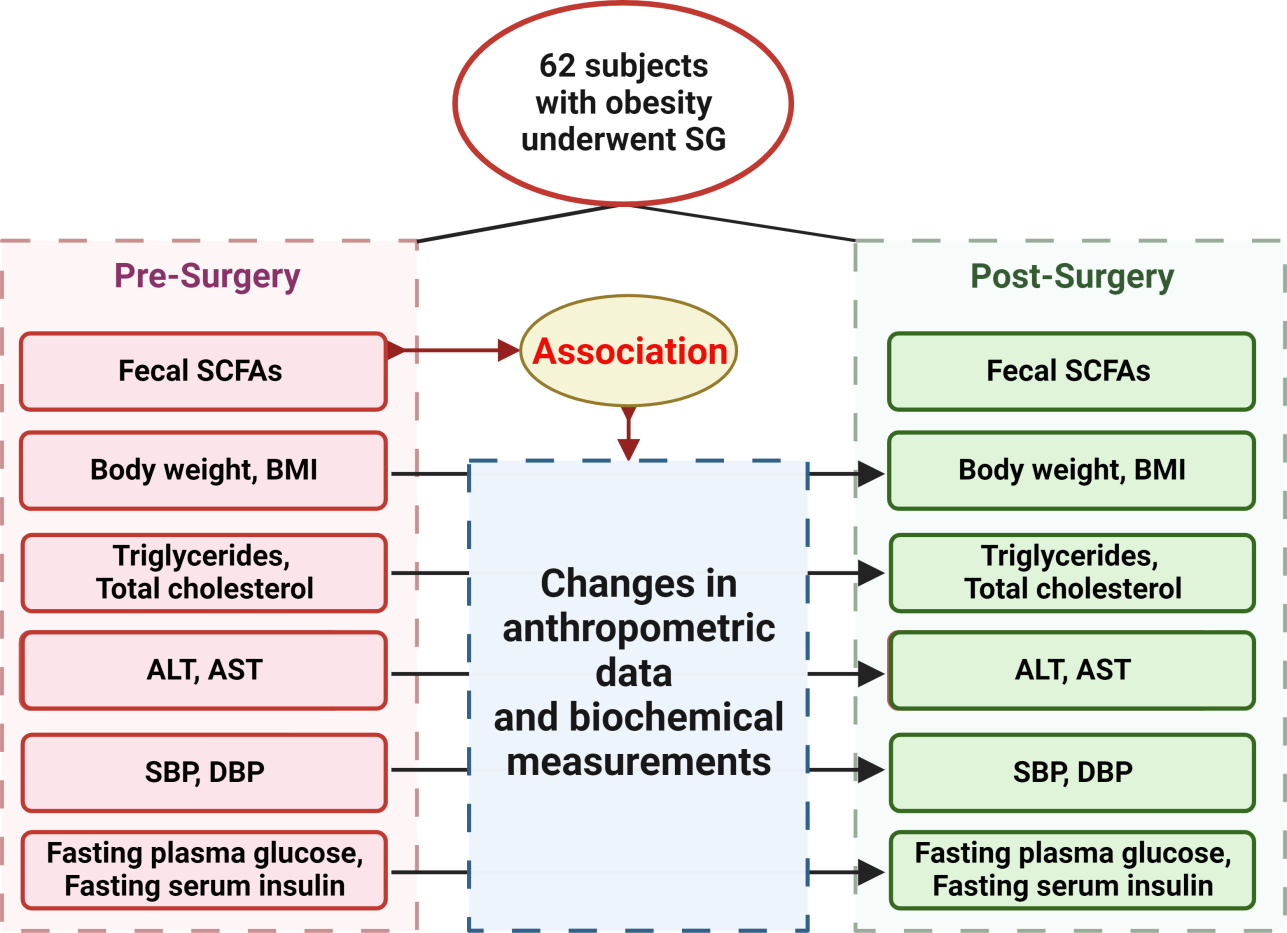
The flow diagram of the study protocol.

62 participants were included in our study initially, and 42, 36, 31 of the participants attended follow-up visit at 1, 3, 6 months after SG, respectively, with anthropometric data, blood and fecal samples. The study was approved by the Institutional Review Board of the Ruijin Hospital, Shanghai Jiao Tong University School of Medicine and a written informed consent was obtained from each participant. **Figure 1** showed the flow diagram of the study protocol.

### 2.2 Fecal short-chain fatty acid analysis

The SCFA concentration was determined using ultra-high performance liquid chromatography-tandem mass spectrometry (UHPLC-MS). A Waters ACQUITY-Ultra High Performance liquid chromatography system (Waters Corp, Milford, USA) coupled with Q Exactive mass spectrometry (Thermo Fisher Scientific, Rockford, IL, USA) was used in negative ion mode. A 5 μL aliquot of each sample was injected into a BEH C18 column (2.1 mm×100 mm, 1.7 μm, Waters Corp, Milford, CT, USA). The Mobile phases were 0.1% formic acid in H2O (phase A) and 0.1% formic acid in acetonitrile (phase B). The gradient started with 15% B, increased to 27% B within 4 min and arrived to 42% at 8 min. Then it increased to 100% in 0.5 min and kept for 3 min. Finally, it returned to the initial 15% B and was stable for 2.5 minutes. The flow rate was 0.35 mL/min and the column temperature was at 40°C. The MS capillary temperature was 300°C with the auxiliary air heating temperature set at 350°C. The sheath gas and auxiliary gas flow rate were set as 45 and 10 units and the spray voltage was 3 KV. Full scan resolution was set as 70K, m/z scan range was 80-1200 Dalton. Thermo Scientific Xcalibur 4.1.31.9 was used for data acquisition.

### 2.3 Clinical parameter measurements

Blood samples were collected after fasting for at least 12 hours, and analyzed for fasting plasma glucose, fasting serum insulin, hemoglobin A1c (HbA1c), alanine aminotransferase (ALT), alanine aminotransferase (ALT), triglycerides, and total cholesterol. Fasting glucose, ALT, AST, triglycerides, and total cholesterol were measured using an autoanalyser (Beckman Coulter AU5800). Fasting insulin was measured using a double antibody radioimmunoassay (DSL, Webster). HbA1c was measured by high-pressure liquid chromatography. Insulin resistance index (homeostasis model assessment of insulin resistance, HOMA-IR) was defined as fasting insulin (IU/mL) × fasting glucose (mmol/L)/22.5, and pancreatic β-cell function index (homeostasis model assessment of β-cell function, HOMA-β) was defined as 20 × fasting insulin (IU/mL)/(fasting glucose (mmol/L) − 3.5).

### 2.4 Statistical analysis

Differences in fecal SCFAs levels and proportions before surgery and at different time-points after surgery were calculated by the paired samples Wilcoxon signed rank test. A P value < 0.05 was considered statistically significant.

We compared the correlation between percent changes ((pre-surgery values-post-surgery values)/pre-surgery values*100) 6 months after bariatric surgery in fecal SCFAs and clinical indices, analyzed by the Spearman correlation test. A P value < 0.05 was considered statistically significant. The correlation heatmap was drawn by GraphPad Prism 9.5.1.

A mixed-effect linear model was employed to discern the impact of bariatric surgery on body weight and metabolic outcomes. More specifically, metabolic measurements, encompassing body weight, BMI, lipid profiles, liver function, blood pressure, and glycemic parameters, were designated as dependent variables. The bariatric surgery was treated as the independent variable, while baseline metabolic measurements and individual basic profiles, such as age and gender, were incorporated as covariates. To mitigate bias introduced by repeated measurements of each participant pre- and post-surgery, individual IDs were treated as random effects.

The same model configured as above was employed to estimate the adjusted effect of bariatric surgery on metabolic outcomes within the population stratified by SCFA groups (with the median serving as the cutoff point for each SCFA).

A similar model with the addition of an interaction term was used to evaluate whether pre-surgery SCFA concentration, as determined by pre-surgery fecal concentration (with the median serving as the cutoff point for each SCFA), modified the effect of bariatric surgery on metabolic outcomes.

All of the calculations above were employed by IBM SPSS Statistics 25.0.

## 3 Results

### 3.1 Clinical characteristics before sleeve gastrectomy

**Table 1** summarized the clinical characteristics of recruited participants before SG. In total, 21 men and 41 women with a mean age of 30.45 years old and mean BMI of 43.35 kg/m2 were included in the analyses, and 42, 36, 31 participants had a follow-up visit at 1, 3, 6 months after SG surgery, respectively.

**Table 1:** Clinical characteristics of recruited participants before sleeve gastrectomy.

Clinical characteristics	Basal values
	Mean ± SD
**Sex (M/F)**	21/41
**Age (Years)**	30.45 ± 7.77
**Weight (Kg)**	122.28 ± 25.05
**BMI (Kg/m^2^)**	43.35 ± 7.93
**SBP (mmHg)**	143.05 ± 18.25
**DBP (mmHg)**	88.18 ± 14.38
**Fasting plasma glucose (mmol/L)**	6.63 ± 2.15
**Fasting serum insulin (uIU/mL)**	26.41 ± 17.56
**HOMA-IR**	8.15 ± 7.24
**HOMA-B**	217.63 ± 161.49
**HbA1c (%)**	6.77 ± 1.59
**ALT (IU/L)**	62.10 ± 40.26
**AST (IU/L)**	39.95 ± 29.54
**Triglycerides (mmol/L)**	2.04 ± 1.21
**Total cholesterol (mmol/L)**	4.68 ± 0.93

### 3.2 Fecal SCFA levels and proportions before and after SG

We examined the changes in the concentrations of fecal SCFAs before and at 1, 3, 6 months after SG treatment (**Table 2**). Among them, acetic acid levels were significantly decreased at 3 time points after SG. Propionic acid levels were significantly decreased at 3, 6 months after SG and showed decreasing trend at 1 month after SG. Butyric acid levels were significantly decreased at 1, 3 months after SG, but rebounded at 6 months after SG. Valeric acid levels, one of minor straight SCFAs, were increased at 3 months after SG. Two main branched SCFAs, isobutyric acid and isovaleric acid, were significantly increased at 1 and 3 months after SG treatment. We also calculated the total concentration of the subgroups of SCFAs. In general, total levels of three major straight SCFAs (acetic-, propionic-, and butyric acids) were lowered at three time points after SG, while total levels of major branched SCFAs (isobutyric- and isovaleric-acids) were raised at 1 months and 3 months after SG.

**Table 2:** Fecal short-chain fatty acid levels and proportions before and at 1 month, 3 months, 6 months after sleeve gastrectomy.

	Basal (n=62)	1 month (n=42)	3 months (n=36)	6 months (n=31)	Basal vs 1month	Basal vs 3months	Basal vs 6months
	Median (P25, P75)	Median (P25, P75)	Median (P25, P75)	Median (P25, P75)	P value	P value	P value
Raw values (umol/g fecal weight)							
**Acetic acid**	48.61 (39.33, 62.45)	36.82 (29.95, 47.53)	39.80 (21.62, 55.51)	37.37 (29.35, 55.42)	**0.034**	**0.020**	**0.003**
**Propionic acid**	20.41 (15.88, 28.42)	15.56 (11.27, 20.70)	15.82 (8.89, 19.91)	15.59 (10.98, 19.85)	0.057	**0.005**	**<0.001**
**Butyric Acid**	11.77 (7.45, 16.65)	6.92 (4.52, 10.59)	8.34 (4.10, 13.10)	10.98 (6.60, 17.44)	**<0.001**	**0.01**	0.088
**Valeric acid**	0.84 (0.34, 2.27)	0.85 (0.36, 2.00)	1.33 (0.69, 2.48)	2.02 (1.39, 2.92)	0.63	**0.050**	0.096
**Hexanoic acid**	0.23 (0.17, 0.29)	0.23 (0.18, 0.32)	0.22 (0.20, 0.28)	0.25 (0.18, 0.44)	0.639	0.48	0.189
**Isobutyric acid**	1.16 (0.52, 1.90)	1.79 (0.96, 2.68)	1.35 (0.83, 2.38)	1.72 (1.16, 2.32)	**0.020**	**0.043**	0.147
**Isovaleric acid**	0.90 (0.41, 1.49)	1.55 (0.77, 2.19)	1.28 (0.71, 2.12)	1.44 (0.92, 2.09)	**0.039**	**0.011**	0.065
**Major straight SCFAs**	88.33 (65.68, 114.20)	63.69 (47.58, 87.13)	68.27 (41.99, 83.46)	67.18 (47.14, 86.81)	**0.013**	**0.012**	**<0.001**
**Major branched SCFAs**	2.04 (0.95, 3.39)	3.33 (1.77, 5.05)	2.66 (1.46, 4.53)	3.14 (2.09, 4.39)	**0.02**	**0.023**	0.104
**Total SCFAs**	92.28 (67.88, 118.28)	65.52 (50.61, 86.49)	73.44 (43.68, 88.33)	74.14 (52.89, 93.00)	**0.020**	**0.025**	**0.002**
Proportions (%)							
**Acetic acid**	55.52 (50.97, 61.07)	59.65 (53.18, 64.62)	57.19 (51.82, 59.95)	55.94 (50.17, 59.94)	0.413	0.572	0.638
**Propionic acid**	25.02 (19.80, 29.77)	24.06 (20.83, 28.18)	23.28 (19.36, 25.86)	20.77 (17.62, 24.67)	0.578	0.093	0.055
**Butyric Acid**	13.14 (10.75, 16.88)	9.87 (7.57, 13.52)	12.78 (9.52, 15.57)	15.20 (12.61, 18.74)	**0.010**	0.226	0.695
**Valeric acid**	0.95 (0.49, 2.18)	1.29 (0.48, 3.30)	2.93 (1.04, 3.78)	3.04 (1.86, 4.64)	**0.049**	**<0.001**	**0.001**
**Hexanoic acid**	0.27 (0.20, 0.41)	0.35 (0.29, 0.53)	0.32 (0.25, 0.56)	0.39 (0.29, 0.60)	**0.040**	**0.040**	**0.025**
**Isobutyric acid**	1.37 (0.75, 1.80)	2.55 (1.58, 3.36)	2.68 (1.39, 3.52)	2.69 (1.07, 3.52)	**<0.001**	**<0.001**	**<0.001**
**Isovaleric acid**	1.14 (0.50, 1.38)	2.00 (1.35, 2.99)	2.19 (1.08, 3.37)	2.18 (0.85, 3.27)	**<0.001**	**<0.001**	**<0.001**
**Major straight SCFAs**	96.10 (93.61, 97.71)	93.54 (90.45, 96.08)	91.20 (89.52, 95.82)	92.17 (87.61, 95.64)	**<0.001**	**<0.001**	**<0.001**
**Major branched SCFAs**	2.54 (1.35, 3.17)	4.75 (2.96, 6.34)	4.90 (2.46, 6.89)	4.85 (1.94, 6.79)	**<0.001**	**<0.001**	**<0.001**

Major straight SCFA concentration was the sum of acetic acid, propionic acid, and butyric acid. Major branched SCFA concentration was the sum of isobutyric acid and isovaleric acid. Total SCFA concentration was the sum of all SCFAs. The proportion is given as the percentage of total SCFAs. The differences between variables before and after SG were employed by Paired Samples Wilcoxon Signed Rank Test. Significance denoted with p-value < 0.05 (bolded).

The proportion of each SCFA could provide additional information of the relative distribution in total SCFAs. We found that acetic acid, propionic acid and butyric acid were three major SCFAs and accounted for 55.52%, 25.02% and 13.14% of the total SCFAs in subjects before SG surgery, respectively, while the other four detected SCFAs accounted for 0.27% to 1.37% of the total SCFAs, which were overall consistent with previous reports (22, 23). After SG treatment, the proportions of acetic acid did not change significantly, and propionic acid proportions were gradually lowered, while butyric acid proportions were lowered at 1 month and rebounded at 3 and 6 months after SG, which were overall consistent with the changes in absolute concentrations. The other four minor SCFAs (including two minor straight SCFAs and two branched SCFAs) were relatively increased after SG treatment at all the 3 time points after SG. Taken together, our data demonstrate that three major SCFAs proportions were lowered, while four minor SCFAs proportions were raised at 1, 3, 6 months after SG treatment.

### 3.3 Correlations between changes in fecal SCFAs and clinical parameters

**Figure 2:**
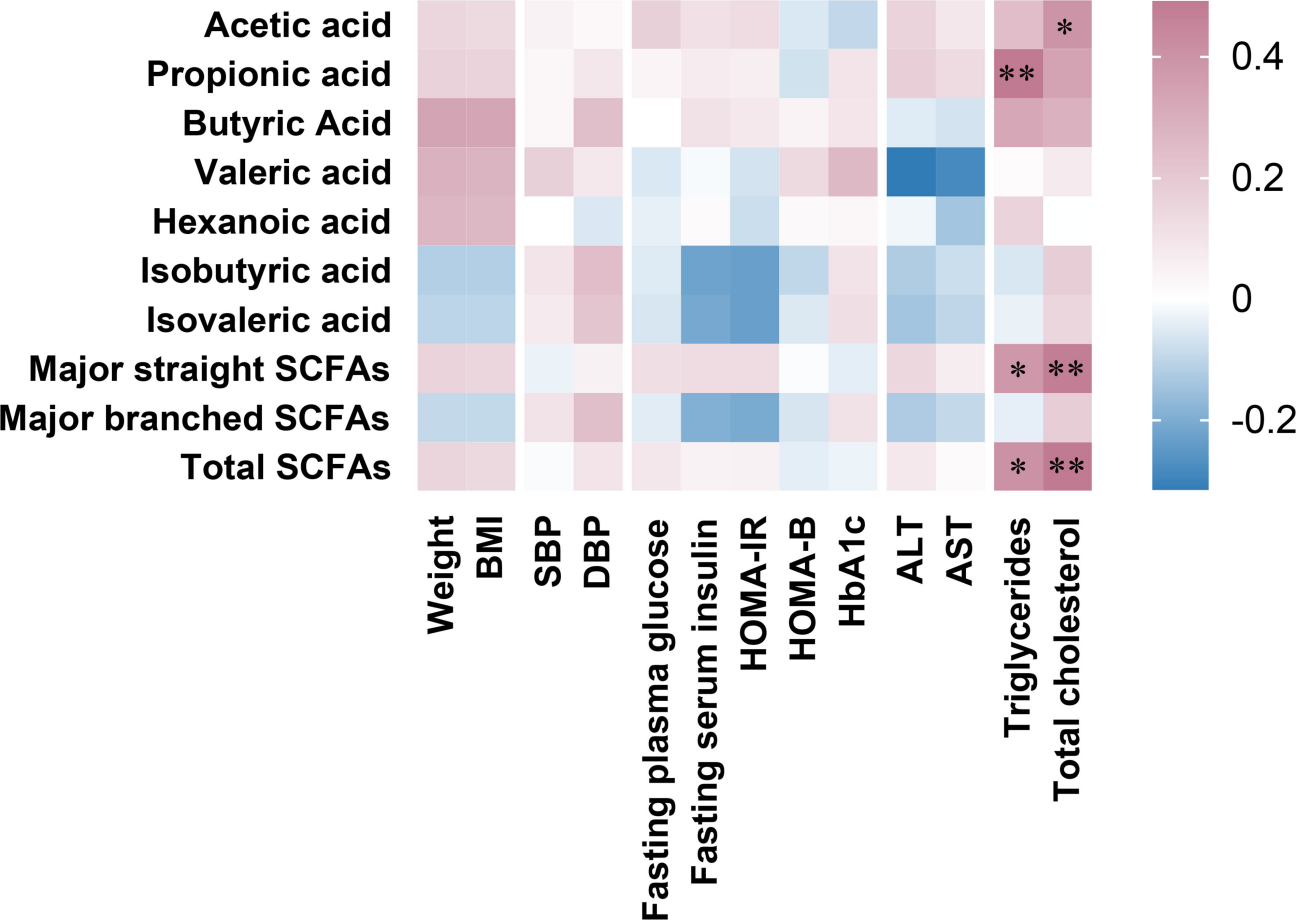
Associations between percent changes ((pre values-post values)/pre values*100) at 6 months after sleeve gastrectomy in fecal SCFAs and clinical parameters. Major straight SCFA concentration was the sum of acetic acid, propionic acid, and butyric acid, major branched SCFA concentration was the sum of isobutyric acid and isovaleric acid, and total SCFA concentration was the sum of all SCFAs. Spearman’s rank correlation coefficients depict positive (red) or inverse (blue) relationship with significance reported with asterisks “*” (p < 0.05) and “**” (p< 0.01).

**Figure 2** revealed the correlations between the percent changes in fecal SCFAs and clinical parameters after 6 months of SG treatment. We found that changes in propionic acid, total major straight SCFAs and total SCFAs were positively correlated with changes in serum triglycerides. Changes in acetic acid, total major straight SCFAs and total SCFAs were positively correlated with changes in serum total cholesterol.

### 3.4 The impact of pre-surgery fecal SCFAs on metabolic responses to SG treatment

We investigated the adjusted changes in clinical indices 6 months after SG using linear mixed model, and found that variables of obesity (**Table 3**), lipid profiles (**Table 4**), liver function (**Table 5**), blood pressure (**Supplementary Table 1**), and glucose metabolism (**Supplementary Table 2**) were significantly improved by SG at 6 months after surgery, indicating a sustainable metabolic improvement after SG intervention.

**Table 3:** Changes in body weight and BMI from baseline to six months after sleeve gastrectomy in all participants and in the subgroups stratified by pre-surgery SCFA levels.

	Body weight (kg)					BMI (Kg/m2)				
	Crude estimate of surgery (95%CI)	P value (surgery)	Estimate of surgery in participants stratified by pre-surgery SCFA groups (95%CI)		P value (group × surgery)	Crude estimate of surgery (95%CI)	P value (surgery)	Estimate of surgery in participants stratified by pre-surgery SCFA groups (95%CI)		P value (group × surgery)
			Low group (<median)	High group (≥median)				Low group (<median)	High group (≥median)	
All participants	-27.79 (-30.67 to -24.92)	**<0.001**				-9.91 (-10.96 to -8.85)	**<0.001**			
Subgroups divided by										
**Acetic acid**			-26.27 (-30.63 to -21.92)	-29.22 (-33.40 to -25.03)	0.312			-9.48 (-11.14 to -7.83)	-10.31 (-11.82 to -8.80)	0.441
**Propionic acid**			-26.03 (-29.81 to -22.25)	-29.45 (-34.13 to -24.76)	0.241			-9.47 (-10.87 to -8.07)	-10.32 (-12.03 to -8.61)	0.428
**Butyric Acid**			-27.11 (-30.63 to -23.59)	-28.43 (-33.28 to -23.58)	0.654			-9.64 (-10.96 to -8.31)	-10.16 (-11.93 to -8.39)	0.627
**Valeric acid**			-27.48 (-30.97 to -23.99)	-28.08 (-33.00 to -23.17)	0.837			-9.87 (-11.16 to -8.57)	-9.95 (-11.75 to -8.15)	0.939
**Hexanoic acid**			-24.09 (-28.52 to -19.67)	-31.26 (-34.66 to -27.86)	**0.01**			-8.82 (-10.47 to -7.18)	-10.93 (-12.22 to -9.63)	**0.041**
**Isobutyric acid**			-27.46 (-31.18 to -23.74)	-28.10 (-32.72 to -23.49)	0.827			-10.06 (-11.44 to -8.67)	-9.77 (-11.46 to -8.08)	0.792
**Isovaleric acid**			-27.93 (-31.65 to -24.20)	-27.67 (-32.30 to -23.03)	0.929			-10.18 (-11.55 to -8.82)	-9.65 (-11.36 to -7.94)	0.622
**Major straight SCFAs**			-27.17 (-30.65 to -23.69)	-28.37 (-33.21 to -23.53)	0.683			-9.81 (-11.15 to -8.47)	-10.00 (-11.76 to -8.25)	0.856
**Major branched SCFAs**			-27.93 (-31.65 to -24.20)	-27.67 (-32.30 to -23.03)	0.929			-10.18 (-11.55 to -8.82)	-9.65 (-11.36 to -7.94)	0.622
**Total SCFAs**			-27.17 (-30.65 to -23.69)	-28.37 (-33.21 to -23.53)	0.683			-9.81 (-11.15 to -8.47)	-10.00 (-11.76 to -8.25)	0.856

Changes from baseline to six months in all participants and in different subgroups (with the median serving as the cutoff point for each SCFA) were expressed as estimated marginal means (95%CI), employed by mixed-effect linear model, where sleeve gastrectomy was treated as the independent variable, which adjusted for age, gender, and baseline values, and individual IDs were treated as random effects. The p-value corresponding to interaction of group by surgery on variables were examined by a similar mixed-effect linear model with the addition of an interaction term of group by surgery. Significance denoted with p-value < 0.05 (bolded).

**Table 4:** Changes in serum triglycerides and total cholesterol from baseline to six months after sleeve gastrectomy in all participants and in the subgroups stratified by pre-surgery SCFA levels.

	Triglycerides (mmol/L)					Total cholesterol (mmol/L)				
	Crude estimate of surgery (95%CI)	P value (surgery)	Estimate of surgery in participants stratified by pre-surgery SCFA groups (95%CI)		P value (group × surgery)	Crude estimate of surgery (95%CI)	P value (surgery)	Estimate of surgery in participants stratified by pre-surgery SCFA groups (95%CI)		P value (group × surgery)
			Low group (<median)	High group (≥median)				Low group (<median)	High group (≥median)	
All participants	-0.83 (-1.09 to -0.57)	**<0.001**				-0.49 (-0.78 to -0.21)	**<0.001**			
Subgroups divided by										
**Acetic acid**			-0.61 (-0.91 to -0.32)	-1.03 (-1.48 to -0.59)	0.116			-0.37 (-0.69 to -0.05)	-0.61 (-1.08 to -0.13)	0.405
**Propionic acid**			-0.77 (-1.10 to -0.44)	-0.88 (-1.32 to -0.44)	0.677			-0.49 (-0.96 to -0.02)	-0.50 (-0.89 to -0.11)	0.979
**Butyric Acid**			-0.69 (-1.00 to -0.37)	-0.96 (-1.41 to -0.51)	0.297			-0.50 (-1.00 to -0.01)	-0.48 (-0.84 to -0.13)	0.939
**Valeric acid**			-0.90 (-1.29 to -0.52)	-0.76 (-1.17 to -0.35)	0.588			-0.61 (-1.19 to -0.04)	-0.38 (-0.60 to -0.17)	0.424
**Hexanoic acid**			-0.54 (-0.79 to -0.29)	-1.10 (-1.55 to -0.65)	**0.031**			-0.65 (-1.21 to -0.10)	-0.34 (-0.57 to -0.10)	0.273
**Isobutyric acid**			-1.08 (-1.44 to -0.73)	-0.59 (-1.00 to -0.18)	0.061			-0.63 (-1.19 to -0.07)	-0.36 (-0.56 to -0.16)	0.343
**Isovaleric acid**			-0.95 (-1.26 to -0.64)	-0.71 (-1.16 to -0.26)	0.367			-0.52 (-1.05 to 0.001)	-0.46 (-0.71 to -0.21)	0.837
**Major straight SCFAs**			-0.73 (-1.04 to -0.43)	-0.92 (-1.38 to -0.46)	0.48			-0.58 (-1.08 to -0.08)	-0.41 (-0.74 to -0.08)	0.541
**Major branched SCFAs**			-0.95 (-1.26 to -0.64)	-0.71 (-1.16 to -0.26)	0.367			-0.52 (-1.05 to 0.001)	-0.46 (-0.71 to -0.21)	0.837
**Total SCFAs**			-0.73 (-1.04 to -0.43)	-0.92 (-1.38 to -0.46)	0.48			-0.58 (-1.08 to -0.08)	-0.41 (-0.74 to -0.08)	0.541

Changes from baseline to six months in all participants and in different subgroups (with the median serving as the cutoff point for each SCFA) were expressed as estimated marginal means (95%CI), employed by mixed-effect linear model, where sleeve gastrectomy was treated as the independent variable, which adjusted for age, gender, and baseline values, and individual IDs were treated as random effects. The p-value corresponding to interaction of group by surgery on variables were examined by a similar mixed-effect linear model with the addition of an interaction term of group by surgery. Significance denoted with p-value < 0.05 (bolded).

**Table 5:** Changes in liver function from baseline to six months after sleeve gastrectomy in all participants and in the subgroups stratified by pre-surgery SCFA levels.

	ALT (IU/L)					AST (IU/L)				
	Crude estimate of surgery (95%CI)	P value (surgery)	Estimate of surgery in participants stratified by pre-surgery SCFA groups (95%CI)		P value (group × surgery)	Crude estimate of surgery (95%CI)	P value (surgery)	Estimate of surgery in participants stratified by pre-surgery SCFA groups (95%CI)		P value (group × surgery)
			Low group (<median)	High group (≥median)				Low group (<median)	High group (≥median)	
All participants	-48.16 (-58.11 to -38.21)	**<0.001**				-27.35 (-36.77 to -17.94)	**<0.001**			
Subgroups divided by										
**Acetic acid**			-50.60 (-65.64 to -35.56)	-45.88 (-60.96 to -30.79)	0.642			-33.93 (-52.85 to -15.02)	-21.19 (-28.89 to -13.49)	0.181
**Propionic acid**			-53.00 (-67.96 to -38.04)	-43.63 (-58.55 to -28.70)	0.354			-35.80 (-54.60 to -17.00)	-19.44 (-26.86 to -12.01)	0.085
**Butyric Acid**			-63.20 (-78.98 to -47.42)	-34.06 (-46.04 to -22.08)	**0.003**			-38.67 (-57.40 to -19.93)	-16.75 (-23.17 to -10.33)	**0.019**
**Valeric acid**			-52.40 (-67.45 to -37.35)	-44.19 (-59.19 to -29.19)	0.418			-29.40 (-38.37 to -20.43)	-25.44 (-43.01 to -7.86)	0.68
**Hexanoic acid**			-43.67 (-59.59 to -27.75)	-52.38 (-66.55 to -38.20)	0.39			-25.20 (-33.68 to -16.72)	-29.38 (-47.17 to -11.58)	0.664
**Isobutyric acid**			-41.07 (-56.40 to -25.73)	-54.81 (-69.22 to -40.40)	0.173			-20.87 (-30.96 to -10.77)	-33.44 (-50.17 to -16.71)	0.188
**Isovaleric acid**			-43.93 (-59.42 to -28.45)	-52.13 (-66.66 to -37.59)	0.419			-21.93 (-31.93 to -11.94)	-32.44 (-49.32 to -15.55)	0.272
**Major straight SCFAs**			-59.93 (-76.09 to -43.77)	-37.13 (-49.74 to -24.51)	**0.022**			-38.07 (-56.85 to -19.28)	-17.31 (-23.87 to -10.75)	**0.027**
**Major branched SCFAs**			-43.93 (-59.42 to -28.45)	-52.13 (-66.66 to -37.59)	0.419			-21.93 (-31.93 to -11.94)	-32.44 (-49.32 to -15.55)	0.272
**Total SCFAs**			-59.93 (-76.09 to -43.77)	-37.13 (-49.74 to -24.51)	**0.022**			-38.07 (-56.85 to -19.28)	-17.31 (-23.87 to -10.75)	**0.027**

Changes from baseline to six months in all participants and in different subgroups (with the median serving as the cutoff point for each SCFA) were expressed as estimated marginal means (95%CI), employed by mixed-effect linear model, where sleeve gastrectomy was treated as the independent variable, which adjusted for age, gender, and baseline values, and individual IDs were treated as random effects. The p-value corresponding to interaction of group by surgery on variables were examined by a similar mixed-effect linear model with the addition of an interaction term of group by surgery. Significance denoted with p-value < 0.05 (bolded).

To investigate whether different pre-surgery SCFAs could impact metabolic responses to SG treatment, we classified the subjects with obesity into low and high-SCFA subgroups based on the median concentrations of each SCFA. Interestingly, we found that high pre-surgery fecal hexanoic acid group showed a better effect of SG surgery on lowering body weight (low vs high -24.09 (95%CI -28.52 to -19.67) vs -31.26 (95%CI -34.66 to -27.86)kg), BMI (low vs high -8.82 (95%CI -10.47 to -7.18) vs -10.93 (95%CI -12.22 to -9.63)kg/m2) (**Table 3**) and serum triglycerides (low vs high -0.54 (95%CI -0.79 to -0.29) vs -1.10 (95%CI -1.55 to -0.65) mmol/L) (**Table 4**) than low pre-surgery fecal hexanoic acid group. Three fecal SCFA components were found to impact the effects of SG on improving liver function. Participants of lower pre-surgery fecal butyric acid or total major straight SCFAs, total SCFAs were observed a better effect of SG on lowering ALT (butyric acid, low vs high, -63.20 (95%CI -78.98 to -47.42) vs -34.06 (95%CI -46.04 to -22.08) IU/L; total major straight SCFAs, total SCFAs, low vs high -59.93(95%CI -76.09 to -43.77) vs -37.13(95%CI -49.74 to -24.51) IU/L) and AST (butyric acid, low vs high, -38.67 (95%CI -57.40 to -19.93) vs -16.75(95%CI -23.17 to -10.33) IU/L; total major straight SCFAs, total SCFAs, low vs high, -38.07 (95%CI -56.85 to -19.28) vs -17.31(95%CI -23.87 to -10.75) IU/L) (**Table 5**). No significance was found in the impact of baseline fecal SCFAs on blood pressure (**Supplementary Table 1**) or parameters of glucose metabolism (**Supplementary Table 2**).

## 4 Discussion

Metabolic benefits produced by bariatric surgery is a complex and multifactorial process, requiring studies to understand the factors involved in and impacting the therapeutic effects. The SCFAs are metabolites from fiber fermentation by gut microbiota, and contribute to the regulation of host health or diseases (25). However, whether SCFAs are affected by SG surgery and impact its therapeutic effects are largely unknown. The present study aimed to comprehensively reveal the profile changes of fecal SCFAs before and in different stages after SG surgery, and investigate the impact of fecal SCFAs’ composition on therapeutic effects of SG treatment.

SCFAs are monocarboxylic acids with one carboxyl group attached to an alkyl group, consisting of 2–6 carbons and comprise of seven types: acetic acid (C2), propionic acid (C3), butyric acid (C4), isobutyric acid (IC4), valeric acid (C5), isovaleric acid (IC5), and hexanoic acid (C6) (26). Straight SCFAs include acetic acid, propionic acid, butyric acid, valeric acid and hexanoic acid, and branched SCFAs are isobutyric acid and isovaleric acid. The most abundant straight SCFAs are acetic, propionic, and butyric acids, and are main catabolic end-products from carbohydrate bacterial fermentation (27–29). Hexanoic and valeric acids account for a small amount of fecal straight SCFAs and have not been proven to be strictly produced by the microbiota (30). Colonic fermentation of the branched-chain amino acids valine and leucine leads to the production of the branched SCFAs isobutyric and isovaleric acids (30, 31).

This study revealed for the first time the profile changes in fecal SCFAs before and in different stages (1month, 3 months and 6 months) after SG in Chinese subjects with obesity. We consistently found that the three straight SCFAs, acetate, propionate and butyrate, account for the major percentage in the gut of the subjects with obesity while after SG treatment, a decrease in the two main straight SCFAs (acetate, propionate), was detected at 3 to 6 months, which were consistent with the decreases in acetate and propionate at 4 to 12 months after surgeries in reports of the combined analysis consisting of both RYGB and SG surgery types (22, 23). Notably, butyric acids transiently decreased at 1 to 3 months after SG, but rebounded at 6 months after SG, which was different with the findings in previous studies using subjects mainly from RYGB and a small sample size of SG (22, 23). Actually, the two surgery types RYGB and SG does not have the same effects on gut microbiota in subjects with obesity (32). As butylates are derived from fiber fermentation by gut microbiota, the different impact of bariatric surgery types on gut microbiota may explain the different butyrate changes in RYGB or SG (32). Besides, an increase in the absolute or proportions of the minor SCFAs, such as isobutyric acid and isovaleric acid, were consistently observed at different time points after SG. Although few studies report the function of these minor SCFAs, our results may suggest their potential role in metabolic regulation.

We found the changes of acetic acid and propionic acid 6 months after SG were positively correlated with the changes of serum total cholesterol and triglycerides, respectively. As reported in a human study, fasting plasma acetate is positively associated with the degree of adiposity, which is interpreted as acetate increasing the source of energy that ends up being stored as lipids (33). In addition, cholesterol can be synthesized from radiolabeled acetate in rat liver (34). Therefore, the positive correlation between acetic acid and serum total cholesterol might be a result of acetate increasing the source of cholesterol biosynthesis in liver. Propionic acid has been also reported to positively correlated with total body fat, visceral fat, and subcutaneous fat in a human study (33). Another study in zebrafish reveals that sodium propionate exposure for 3 months increases triglycerides, total cholesterol and blood glucose (35). Propionic acid can promote sympathetic outflow via GPR41 (36), increasing lipolysis in WAT and enhancing VLDL-TG production by the liver (37), which support the positive relationship between changes in propionic acid and serum triglycerides in our study.

We found that high pre-surgery fecal hexanoic acid group showed a better effect of SG treatment on lowering body weight, BMI and triglycerides than low pre-surgery fecal hexanoic acid group. Hexanoic acid, a straight-chain saturated fatty acid of C6, accounts for a small amount of SCFAs and is poorly studied. In a study performed on chick embryos and HepG2 hepatocytes, hexanoic acid is found to decrease insulin and triiodothyronine-induced fatty acid synthase (FASN) expression and activity (38), which is a key enzyme in *de novo* lipogenesis (39). In another study performed in the HepG2 hepatoma cell line, hexanoic acid promoted basal and insulin-induced phosphorylation of the Akt-mTOR axis, maintaining a balance of lipid metabolism and potentially constituting an effective tool to manage liver steatosis and hepatic insulin resistance (40). A human study evaluated the association between fecal fungi and obesity, and found that Eurotiomycetes, which could colonize in the gastrointestinal tract of both obese and non-obese subjects, might modify metabolic phenotype in obese subjects (41). Obese subjects with Eurotiomycetes <1% had a more pronounced dyslipidemic profile, increased fasting triglycerides, increased total cholesterol and increased fasting hyperinsulinemia, compared with obese subjects with Eurotiomycetes >1%. The authors also performed a plasma metabolomics profile and found that relative abundance of Eurotiomycetes was positively associated with plasma hexanoic acid. Although it remains to be confirmed whether hexanoic acids are catabolic end-products from Eurotiomycetes and whether hexanoic acids directly modify the metabolic phenotype in obese subjects, these findings support that hexanoic acid might be a potential factor impacting SG’s effects on lowering body weight, BMI and triglycerides, and more researches on the direct effects of hexanoic acid on body weight and lipid metabolism are needed.

When it comes to the impact of pre-surgery fecal SCFAs on the liver function improvements after SG surgery, low baseline fecal butyric acid was found to have a better effect of SG on improving liver function. Fasting plasma butyrate has been proven to be positively associated with the degree of adiposity, hepatic fat and an increase in hepatic *de novo* lipogenesis in adolescents with obesity (33). In the liver, butyrate can be converted to acetyl-CoA, which are substrates for hepatic *de novo* lipogenesis (42). Hepatic fat is closely related with inflammation and hepatocyte damage (43). These findings suggest that subjects with obesity having low baseline fecal butyric acid might have a reduced potential of hepatic fat production, possibly making them more responsive to SG treatment in improving liver function. However, there are also studies reporting that butyric acid attenuated steatohepatitis (44). Daily intragastric administration with sodium butyrate at 200 mg/kg body weight for 8 weeks lowers the levels of triglycerides and cholesterol in liver and improve liver function in mice fed a high-fat diet (44). Therefore, butyric acid can be beneficial after long term and high dose application, and the mechanism underlying fecal butyrate influencing liver function improvement after bariatric surgery remains to be researched.

In our study, we revealed for the first time the impact of fecal SCFAs on therapeutic effects of SG surgery in subjects with obesity. However, there remains unresolved questions to be answered: 1) the range of fecal SCFAs that predicts a better therapeutic effect of SG should be determined in larger population; 2) only subjects with obesity undergoing SG were included in our study, and whether fecal SCFAs have an influence on therapeutic effects of RYGB is unknown, which is also an interesting topic.

The results presented in this study should also be interpreted with some limitations. For example, our study followed up to 6 months after SG surgery, and the long-term (one year or more) effects could not be determined. Second, in the current study, eating preferences or habits of participants were lacking, which, however, could be an important modifier to bariatric surgery for investigation. Third, the SCFAs levels in the blood could reflect the absorption of SCFAs from intestine and the utilization of SCFAs *in vivo* and likewise affect the SG surgery but were not examined in our study, which also warrants a future comprehensive investigation. Despite of these limitations, our findings may shed lights on the mechanistic researches underlying SCFAs influencing therapeutic effects of bariatric surgery. In addition, applying baseline measurement of fecal SCFAs may help to predict or assess the therapeutic capability of bariatric surgery in patients with obesity.

## 5 Conclusion

In conclusion, we comprehensively revealed the profile changes of fecal SCFAs before and in different stages after SG surgery, uncovered the positive correlations of acetate and propionate changes with lipid post-surgery changes, and suggested that baseline fecal butyric acid and hexanoic acid have an impact on the effect of SG surgery on metabolic improvement. To our best knowledge, this is the first study assessing the impact of fecal SCFAs on metabolic benefits after SG surgery, providing important implications for personalized management of obesity and metabolic disorders by SG surgery.

## Data availability statement

Datasets generated during the current study are available from the corresponding author upon reasonable request.

## Ethics statement

The studies involving humans were approved by Institutional Review Board of the Ruijin Hospital. The studies were conducted in accordance with the local legislation and institutional requirements. The participants provided their written informed consent to participate in this study.

## Author contributions

CS: Investigation, Methodology, Writing – original draft. YRC: Investigation, Methodology, Writing – review & editing. QW: Investigation, Methodology, Writing – review & editing. YS: Data curation, Methodology, Writing – review & editing. HL: Resources, Writing – review & editing. MN: Resources, Writing – review & editing. YFC: Resources, Writing – review & editing. LZ: Resources, Writing – review & editing. JJ: Resources, Writing – review & editing. XY: Resources, Writing – review & editing. YYZ: Resources, Writing – review & editing. XW: Resources, Writing – review & editing. YY: Resources, Writing – review & editing. MY: Resources, Writing – review & editing. NY: Resources, Writing – review & editing. ZC: Resources, Writing – review & editing. YFZ: Writing – review & editing. WG: Writing – review & editing. WW: Writing – review & editing. GN: Writing – review & editing. JW: Writing – review & editing. SZ: Conceptualization, Project administration, Writing – review & editing, Supervision. JH: Conceptualization, Project administration, Writing – review & editing, Supervision. RL: Conceptualization, Project administration, Writing – original draft, Supervision.
