# Supplementary material for: Fecal short chain fatty acids modify therapeutic effects of sleeve gastrectomy
Source: Front Endocrinol (Lausanne). 2023 Nov 7;14:1277035. doi: 10.3389/fendo.2023.1277035 (PMC10663943; doi:10.3389/fendo.2023.1277035)
Supplement: Supplementary file 1 [file DataSheet_1.docx]

Supplementary Material

**Table S1.** Changes in blood pressure from baseline to six months after sleeve gastrectomy in all participants and in the subgroups stratified by pre-surgery SCFA levels. Changes from baseline to six months in all participants and in different subgroups (with the median serving as the cutoff point for each SCFA) were expressed as estimated marginal means (95%CI), employed by mixed-effect linear model, where sleeve gastrectomy was treated as the independent variable, which adjusted for age, gender, and baseline values, and individual IDs were treated as random effects. The p-value corresponding to interaction of group by surgery on variables were examined by a similar mixed-effect linear model with the addition of an interaction term of group by surgery. Significance denoted with p-value < 0.05 (bolded).

|  | **SBP(mmHg)** | | | | | **DBP(mmHg)** | | | | |
| --- | --- | --- | --- | --- | --- | --- | --- | --- | --- | --- |
|  | Crude estimate of surgery (95%CI） | P value (surgery) | Estimate of surgery in participants stratified by pre-surgery SCFA groups (95%CI） | | P value  (group × surgery) | Crude estimate of surgery (95%CI） | P value  (surgery) | Estimate of surgery in participants stratified by pre-surgery SCFA groups (95%CI） | | P value  (group × surgery) |
|  |  |  | Low group(<median) | High group(≥median) |  |  |  | Low group(<median) | High group(≥median) |  |
| **All participants** | -15.10(-21.64 to -8.55) | **<0.001** |  |  |  | -7.45(-13.11 to -1.79) | **0.011** |  |  |  |
| **Subgroups divided by** | |  |  |  |  |  |  |  |  |  |
| **Acetic acid** |  |  | -12.67(-23.58 to -1.75) | -17.38(-25.23 to -9.52) | 0.48 |  |  | -1.87(-10.74 to 7.01) | -12.69(-19.33 to -6.04) | 0.057 |
| **Propionic acid** |  |  | -11.00(-21.78 to -0.22) | -18.94(-26.78 to -11.10) | 0.225 |  |  | -4.27(-12.43 to 3.90) | -10.44(-18.86 to -2.01) | 0.28 |
| **Butyric Acid** |  |  | -11.27(-22.20 to -0.33) | -18.69(-26.42 to -10.96) | 0.264 |  |  | -2.27(-11.26 to 6.72) | -12.31(-19.41 to -5.22) | 0.075 |
| **Valeric acid** |  |  | -13.80(-24.10 to -3.50) | -16.31(-25.63 to -7.00) | 0.707 |  |  | -10.13(-18.28 to -1.98) | -4.94(-13.78 to 3.90) | 0.368 |
| **Hexanoic acid** |  |  | -18.67(-28.38 to -8.95) | -11.75(-21.74 to -1.76) | 0.298 |  |  | -10.33(-18.31 to -2.35) | -4.75(-13.39 to 3.89) | 0.333 |
| **Isobutyric acid** |  |  | -14.87(-24. 57 to -5.16) | -15.31(-24.89 to -5.74) | 0.947 |  |  | -8.47(-16.76 to -0.17) | -6.50(-15.28 to 2.28) | 0.734 |
| **Isovaleric acid** |  |  | -13.67(-23.70 to -3.63) | -16.44(-25.64 to -7.24) | 0.678 |  |  | -6.53(-15.05 to 1.99) | -8.31(-16.65 to 0.03) | 0.758 |
| **Major straight SCFAs** |  |  | -12.27(-23.23 to -1.30) | -17.75(-25.46 to -10.04) | 0.411 |  |  | -2.27(-10.90 to 6.37) | -12.31(-19.38 to -5.24) | 0.074 |
| **Major branched SCFAs** |  |  | -13.67(-23.70 to -3.63) | -16.44(-25.64 to -7.24) | 0.678 |  |  | -6.53(-15.05 to 1.99) | -8.31(-16.65 to 0.03) | 0.758 |
| **Total SCFAs** |  |  | -12.27(-23.23 to -1.30) | -17.75(-25.46 to -10.04) | 0.411 |  |  | -2.27(-10.90 to 6.37) | -12.31(-19.38 to -5.24) | 0.074 |

**Table S2.** Changes in markers of glucose metabolism from baseline to six months after sleeve gastrectomy in all participants and in the subgroups stratified by pre-surgery SCFA levels. Changes from baseline to six months in all participants and in different subgroups (with the median serving as the cutoff point for each SCFA) were expressed as estimated marginal means (95%CI), employed by mixed-effect linear model, where sleeve gastrectomy was treated as the independent variable, which adjusted for age, gender, and baseline values, and individual IDs were treated as random effects. The p-value corresponding to interaction of group by surgery on variables were examined by a similar mixed-effect linear model with the addition of an interaction term of group by surgery. Significance denoted with p-value < 0.05 (bolded).

|  | **Fasting plasma glucose(mmol/L)** | | | | | **Fasting serum insulin(uIU/mL)** | | | | |
| --- | --- | --- | --- | --- | --- | --- | --- | --- | --- | --- |
|  | Crude estimate of surgery (95%CI） | P value (surgery) | Estimate of surgery in participants stratified by pre-surgery SCFA groups (95%CI） | | P value  (group × surgery) | Crude estimate of surgery (95%CI） | P value  (surgery) | Estimate of surgery in participants stratified by pre-surgery SCFA groups (95%CI） | | P value  (group × surgery) |
|  |  |  | Low group(<median) | High group(≥median) |  |  |  | Low group(<median) | High group(≥median) |  |
| **All participants** | -2.25(-2.83 to -1.66) | **<0.001** |  |  |  | -17.80(-23.49 to -12.09) | **<0.001** |  |  |  |
| **Subgroups divided by** | |  |  |  |  |  |  |  |  |  |
| **Acetic acid** |  |  | -2.63(-3.63 to -1.63) | -1.90(-2.66 to -1.15) | 0.23 |  |  | -16.17(-21.63 to -10.72) | -19.23(-29.73 to -8.73) | 0.601 |
| **Propionic acid** |  |  | -2.21(-3.08 to -1.33) | -2.28(-3.17 to -1.39) | 0.906 |  |  | -23.47(-33.91 to -13.03) | -12.66(-18.88 to -6.44) | 0.059 |
| **Butyric Acid** |  |  | -1.78(-2.44 to -1.13) | -2.67(-3.67 to -1.66) | 0.138 |  |  | -15.33(-20.56 to -10.11) | -19.98(-30.52 to -9.45) | 0.425 |
| **Valeric acid** |  |  | -2.04(-2.85 to -1.24) | -2.43(-3.36 to -1.49) | 0.533 |  |  | -16.61(-32.86 to -9.36) | -18.80(-28.37 to -9.21) | 0.708 |
| **Hexanoic acid** |  |  | -2.74(-3.73 to -1.76) | -1.81(-2.83 to -0.79) | 0.121 |  |  | -16.82(-23.23 to -10.41) | -18.65(-28.62 to -8.68) | 0.743 |
| **Isobutyric acid** |  |  | -2.08(-2.94 to -1.21) | -2.39(-3.29 to -1.50) | 0.608 |  |  | -15.61(-22.48 to -8.75) | -19.71(-29.45 to -9.98) | 0.479 |
| **Isovaleric acid** |  |  | -1.85(-2.72 to -0.99) | -2.60(-3.48 to -1.72) | 0.215 |  |  | -15.81(-22.59 to -9.02) | -19.54(-29.31 to -9.77) | 0.518 |
| **Major straight SCFAs** |  |  | -2.18(-3.04 to -1.32) | -2.31(-3.21 to -1.41) | 0.832 |  |  | -17.34(-22.96 to -11.89) | -18.07(-28.56 to -7.58) | 0.915 |
| **Major branched SCFAs** |  |  | -1.85(-2.72 to -0.99) | -2.60(-3.48 to -1.72) | 0.215 |  |  | -15.81(-22.59 to -9.02) | -19.54(-29.31 to -9.77) | 0.518 |
| **Total SCFAs** |  |  | -2.18(-3.04 to -1.32) | -2.31(-3.21 to -1.41) | 0.832 |  |  | -17.34(-22.96 to -11.89) | -18.07(-28.56 to -7.58) | 0.915 |
